# Race/ethnicity-specific associations between breastfeeding information source and breastfeeding rates among U.S. women

**DOI:** 10.1186/s12889-023-15447-8

**Published:** 2023-03-17

**Authors:** Stephanie M. Quintero, Paula D. Strassle, Amalia Londoño Tobón, Stephanie Ponce, Alia Alhomsi, Ana I. Maldonado, Jamie S. Ko, Miciah J. Wilkerson, Anna María Nápoles

**Affiliations:** Division of Intramural Research, National Institute On Minority Health and Health Disparities, National Institute of Health, Bethesda, 11545 Rockville Pike 2WF RM C13, Rockville, MD 20818 USA

**Keywords:** PRAMS, Breastfeeding, Health disparities, Maternal and Infant Health, Race/Ethnicity

## Abstract

**Background:**

Despite evidence of the impact of breastfeeding information on breastfeeding rates, it is unknown if information sources and impact vary by race/ethnicity, thus this study assessed race/ethnicity-specific associations between breastfeeding information sources and breastfeeding.

**Methods:**

We used data from the 2016–2019 Pregnancy Risk Assessment Monitoring System. Race/ethnicity-stratified multinomial logistic regression was used to estimate associations between information source (e.g., family/friends) and breastfeeding rates (0 weeks/none, < 10 weeks, or ≥ 10 weeks; < 10 weeks and ≥ 10 weeks = any breastfeeding). All analyses were weighted to be nationally representative.

**Results:**

Among 5,945,018 women (weighted), 88% reported initiating breastfeeding (≥ 10 weeks = 70%). Information from family/friends (< 10 weeks: aORs = 1.58–2.14; ≥ 10 weeks: aORs = 1.63–2.64) and breastfeeding support groups (< 10 weeks: aORs = 1.31–1.76; ≥ 10 weeks: aORs = 1.42–2.77) were consistently associated with breastfeeding and duration across most racial/ethnic groups; effects were consistently smaller among Alaska Native, Black, and Hispanic women (vs White women). Over half of American Indian and one-quarter of Black women reported not breastfeeding/stopping breastfeeding due to return to school/work concerns.

**Conclusions:**

Associations between breastfeeding information source and breastfeeding rates vary across race/ethnicity. Culturally tailored breastfeeding information and support from family/friends and support groups could help reduce breastfeeding disparities. Additional measures are needed to address disparities related to concerns about return to work/school.

**Supplementary Information:**

The online version contains supplementary material available at 10.1186/s12889-023-15447-8.

## Background

Breastfeeding has many benefits for the infant and breastfeeding person. For example, longer breastfeeding duration can decrease risk of respiratory infections and mortality among infants and decrease risk of breast cancer and cardiovascular disease for the breastfeeding person [1]. Breastfeeding has also been linked to future behaviors such as increased resilience to psychosocial stressors [2] and decreased childhood maltreatment [3]. The American Academy of Pediatrics recommends that breastfeeding people exclusively breastfeed their infants for the first 6 months and continue to breastfeed with the introduction of complementary foods for a year or longer [1]. Despite the known benefits of breastfeeding, significant racial/ethnic disparities exist in the rates of initiation and duration of breastfeeding within the United States (U.S.) For instance, non-Hispanic Black (75% vs 83% of all children born in 2018) and American Indian/Alaska Native people (81% vs. 84% of all children born in 2017) are the least likely to initiate breastfeeding [4, 5]. Non-Hispanic Black (49%), Hispanic/Latino (53%), and multiracial adults (53%) are also less likely to breastfeed for ≥ 6 months, compared to the U.S. national rate (57%) [4, 5].

Multiple interrelated factors likely contribute to racial/ethnic disparities in breastfeeding, including historical, cultural, social, economic, political, and psychosocial factors [6]. Structural factors, such as mode of newborn delivery, socioeconomic status, and return to work, have also influenced breastfeeding rates in the U.S [7, 8]. While many barriers for increasing breastfeeding initiation and duration are structural, breastfeeding education through various information sources is a cost-effective intervention to improve breastfeeding rates overall and among diverse racial/ethnic populations [9–12].

Despite studies suggesting that breastfeeding information impacts breastfeeding rates, to our knowledge, there have been no studies that investigated if the impact of breastfeeding information sources differs across race/ethnicity in terms of improving breastfeeding initiation and duration. Thus, the purpose of this study is to 1) estimate the prevalence of breastfeeding information sources across race/ethnicity, and 2) determine whether the effect of breastfeeding information source on any breastfeeding and breastfeeding duration differs by race/ethnicity. A more nuanced understanding of the impact of specific sources of breastfeeding information among diverse populations could aid in streamlining and tailoring education interventions to decrease racial/ethnic disparities in breastfeeding.

## Methods

### Study population

We used data from the Pregnancy Risk Assessment Monitoring System (PRAMS), a multi-site, population-based surveillance system that samples women 2–6 months after a live birth. PRAMS draws information from each participating state’s birth certificate file and asks questions about maternal health behaviors and experiences before, during, and after pregnancy. Details on the PRAMS study design and methodology have been described elsewhere [13]. For this study, we used the most recent PRAMS survey data, Phase 8, 2016–2019.

All women who reported currently living with their infant were eligible for inclusion. Women were excluded if they were missing race/ethnicity (*n* = 5,349) or reported as “other race” (*n* = 5,071); were < 18 years old or missing age (*n* = 1,762); completed the PRAMS survey < 10 weeks after giving birth (*n* = 112); or had missing data on breastfeeding outcomes (*n* = 5,494) or on breastfeeding information sources (*n* = 9,379). After exclusions, our final study cohort included 116,132 (5,945,018 weighted) individuals who gave birth between 2016 and 2019 from 37 states, Puerto Rico, and New York City. Henceforth, we will refer to PRAMS survey respondents as women, since all individuals included in our analysis self-identified as such. Demographics for women included in the study, stratified by race/ethnicity, are reported in Supplemental Table 1.

### Dependent variable: breastfeeding initiation and duration

Any breastfeeding and breastfeeding duration were captured using the question “How many weeks or months did you breastfeed or feed pumped milk to your new baby?” Any breastfeeding and duration were categorized into one 3-level variable categorized as 1) did not breastfeed or breastfed 0 weeks/none, 2) breastfed < 10 weeks, and 3) breastfed ≥ 10 weeks. Since all women completed the survey > 10 weeks after birth, those who reported “currently still breastfeeding” were categorized as breastfed ≥ 10 weeks. This definition has been used previously [14].

Each PRAMS participating site could include additional questions from a standardized library. These questions expand on topics covered in the core questionnaire. Two additional questions asked: “What were your reasons for stopping breastfeeding?” and “What were your reasons for not breastfeeding your new baby?” Data from these questions (22 sites included the first question and 20 included the second question) were included in analyses. A complete list of the sites and response options are found in the PRAMS documentation [15].

### Independent variable: sources of breastfeeding information

Breastfeeding information sources were captured using the question “Before or after your new baby was born, did you receive information about breastfeeding from any of the following sources (check all that apply)?” Responses included: my doctor; a nurse, midwife, or doula; a breastfeeding or lactation specialist; my baby’s doctor or health care provider; a breastfeeding support group; a breastfeeding hotline or toll-free number; family or friend; and other. Participants were instructed to select all that apply with response options of yes or no to each source. Because a breastfeeding or lactation specialist is often contacted when women are having difficulty breastfeeding (i.e., after breastfeeding initiation), this source was excluded in our modeling analyses but prevalence by race/ethnicity was reported.

### Other variables of interest

Race/ethnicity and other maternal demographics were obtained through birth certificate records and were available in PRAMS. Race identification options were American Indian, Alaska Native, Black, Asian, Native Hawaiian, mixed race, and White. Hispanic ethnicity was captured separately. We combined race/ethnicity into a single variable, in which anyone who identified as Hispanic was included as Hispanic.

Other birth certificate variables of interest involved age at delivery, education (i.e., elementary/some high school, high school degree, some college, and college degree or higher), and prenatal care adequacy (i.e., inadequate, intermediate, adequate, and adequate plus). Adequacy of prenatal care was characterized using the Kotelchuck Index [16] (i.e., Adequacy of Prenatal Care Utilization), which is a summary score based on the timing of initiation of prenatal care and total number of prenatal care visits.

### Statistical analyses

Multivariable multinomial logistic regression was used to estimate the association between breastfeeding information sources (baby’s doctor, personal doctor, family/friends, support group, hotline, nurse/midwife/doula) and breastfeeding initiation/duration (≥ 10 weeks, < 10 weeks, no breastfeeding [reference]), adjusting for age at delivery, education, adequacy of prenatal care (Kotelchuck index). All breastfeeding information sources (besides lactation specialist) were included in the model. Therefore, models estimated the association between receiving information from a specific source (e.g., baby’s doctor), compared to not receiving breastfeeding information from that source with the breastfeeding initiation/duration outcomes, adjusted for the variables listed above and the other information sources. To estimate the associations between breastfeeding information sources and breastfeeding initiation/duration within each racial/ethnic group, separate models were run within each race/ethnicity. Due to the small sample size of women who identified as Native Hawaiian (*n* = 44 unweighted), they were excluded from all modeling.

As a sensitivity analysis, we also assessed the impact of breastfeeding information sources by language (i.e., English- vs. Spanish-speaking) among Hispanic women. Similar to above, we ran two separate multivariable multinomial logistic regression models, one among English-speaking Hispanic women and one among Spanish-speaking Hispanic women, adjusting for age at delivery, education, and adequacy of prenatal care. We also ran a sensitivity analysis where lactation specialist was included as a breastfeeding information source in our models.

All analyses were conducted using SAS version 9.4 using complex survey methods and weighting to obtain national estimates.

## Results

Overall, 88% of women reported any breastfeeding; Black (77%) and American Indian (82%) women were least likely to report any breastfeeding, compared to other groups (89%-100%), Table 1. Among those who reported any breastfeeding (*n* = 5,204,758), Black (57%) and American Indian (61%) women were also least likely to breastfeed ≥ 10 weeks, followed by Hispanic women (64%), compared to other racial/ethnic groups (70%-81%). Native Hawaiian (> 99%), Asian (93%), and Alaska Native (92%) women were the most likely to report any breastfeeding, and Asian and Native Hawaiian women were the most likely to report breastfeeding for ≥ 10 weeks (81% for both groups. Over 90% of both English- (≥ 10 weeks: 56.7%; < 10 weeks: 34.1%) and Spanish-speaking (≥ 10 weeks: 61.5%; < 10 weeks: 30.4%) Hispanic women reported any breastfeeding.

**Table 1:** Any breastfeeding, breastfeeding duration, reasons for not starting breastfeeding (among those reporting they did not breastfeed/breastfed 0 weeks/none only), and reasons for stopping breastfeeding (among those reporting any breastfeeding but < 10 weeks duration), stratified by race/ethnicity, weighted to be site representative, Pregnancy Risk Assessment Monitoring System Phase 8, 2016–2019

	**Overall**		**American Indian**		**AlaskaNative**		**Asian**		**Black**		**Hispanic**		**Native Hawaiian**		**White**		**Multiracial**	
**Any breastfeeding**																		
Yes	5,204,758	(87.5)	30,464	(82.3)	5,555	(91.5)	293,247	(92.6)	655,357	(77.1)	954,835	(91.2)	968	(99.5)	3,138,993	(88.5)	125,338	(89.7)
No	740,260	(12.5)	6,546	(17.7)	514	(8.5)	23,461	(7.4)	195,124	(22.9)	91,723	(8.8)	5	(0.5)	408,433	(11.5)	14,455	(10.3)
**Breastfeeding duration**^**a**^																		
< 10 weeks	1,563,609	(30.0)	12,033	(39.5)	1,473	(26.5)	56,912	(19.4)	280,310	(42.8)	340,377	(35.6)	183	(18.9)	834,960	(26.6)	37,362	(29.8)
≥ 10 weeks	3,641,149	(70.0)	18,432	(60.5)	4,082	(73.5)	236,335	(80.6)	375,047	(57.2)	614,458	(64.4)	785	(81.1)	2,304,033	(73.4)	87,976	(70.2)
**Reasons for not starting**^**b**^																		
Sick/on medicine	44,984	(12.9)	228	(28.8)	N/A^d^		254	(7.1)	11,104	(9.9)	5,352	(14.3)	N/A^d^		27,097	(14.2)	948	(16.7)
Take care of other children	82,408	(23.5)	65	(8.2)	N/A^d^		286	(8.1)	22,314	(19.9)	9,568	(25.6)	N/A^d^		48,867	(25.6)	1,308	(23.1)
Too many household duties	48,399	(13.8)	86	(10.8)	N/A^d^		155	(4.4)	15,171	(13.6)	5,214	(13.9)	N/A^d^		26,623	(14.0)	1,151	(20.3)
Didn’t like it	94,815	(27.1)	264	(33.4)	N/A^d^		639	(18.0)	36,089	(32.3)	9,251	(24.7)	N/A^d^		47,115	(24.8)	1,458	(25.7)
Tried but too hard	56,589	(16.2)	78	(9.9)	N/A^d^		620	(17.5)	20,848	(18.6)	10,769	(28.8)	N/A^d^		23,135	(12.1)	1,138	(20.1)
Didn’t want to	163,751	(46.8)	614	(77.5)	N/A^d^		971	(27.3)	56,698	(50.7)	10,616	(28.4)	N/A^d^		92,298	(48.4)	2,554	(45.1)
Went back to work	66,154	(18.9)	211	(26.6)	N/A^d^		505	(14.2)	23,260	(20.8)	4,785	(12.8)	N/A^d^		36,270	(19.0)	1,123	(19.8)
Went back to school	9,943	(2.8)	187	(23.7)	N/A^d^		232	(6.5)	4,324	(3.9)	289	(0.8)	N/A^d^		4,816	(2.5)	94	(1.7)
Other	77,773	(22.2)	113	(14.3)	N/A^d^		691	(19.6)	17,196	(15.4)	10,125	(27.1)	N/A^d^		47,856	(25.1)	1,792	(31.6)
**Reasons for stopping**^**c**^					N/A^d^								N/A^d^					
Baby had difficulty latching	232,286	(38.7)	1,559	(37.7)	N/A^d^		10,486	(42.4)	33,670	(32.9)	42,987	(39.5)	N/A^d^		138,607	(40.2)	4,956	(34.8)
Breastmilk not enough	230,168	(38.5)	1,320	(32.1)	N/A^d^		11,138	(45.3)	30,183	(29.5)	45,788	(42.3)	N/A^d^		136,081	(39.5)	5,653	(39.8)
Baby not gaining enough weight	102,866	(17.2)	792	(19.2)	N/A^d^		2,878	(11.7)	14,245	(13.9)	16,497	(15.2)	N/A^d^		66,266	(19.2)	2,183	(15.3)
Nipple pain/soreness	152,869	(25.5)	1,404	(34.1)	N/A^d^		5,661	(23.0)	33,284	(32.5)	29,089	(26.8)	N/A^d^		79,475	(23.0)	3,939	(27.7)
Not producing enough milk	344,969	(57.6)	2,491	(60.5)	N/A^d^		14,961	(60.7)	49,920	(48.8)	63,826	(58.7)	N/A^d^		204,420	(59.2)	9,278	(65.2)
Got sick/medical reasons	62,011	(10.4)	494	(12.0)	N/A^d^		2,031	(8.3)	10,714	(10.5)	14,581	(13.4)	N/A^d^		32,837	(9.5)	1,336	(9.5)
Too many household duties	103,157	(17.2)	900	(21.9)	N/A^d^		3,876	(15.8)	20,670	(20.2)	15,433	(14.2)	N/A^d^		59,395	(17.2)	2,866	(20.3)
Felt it was right time to stop	65,227	(10.9)	460	(11.2)	N/A^d^		1,726	(7.0)	14,397	(14.1)	11,722	(10.8)	N/A^d^		35,129	(10.2)	1,776	(12.6)
Went back to work	99,867	(16.7)	655	(16.0)	N/A^d^		4,793	(19.5)	24,404	(23.8)	15,548	(14.3)	N/A^d^		52,617	(15.3)	1,782	(12.6)
Went back to school	15,600	(2.6)	165	(4.0)	N/A^d^		718	(2.9)	4,918	(4.8)	3,369	(3.1)	N/A^d^		5,598	(1.6)	832	(5.9)
Other	115,428	(19.3)	827	(20.1)	N/A^d^		3,791	(15.3)	14,596	(14.3)	18,877	(17.3)	N/A^d^		74,873	(21.7)	2,463	(17.3)

^a^ Among those who reported any breastfeeding

^b^ Among women who reported they did not breastfeed/breastfed 0 weeks/none; sites that asked this question included: AL, AR, IA, IL, KY, LA, ME, MI, MO, MT, NC, ND, NH, PR, RI, SD, TX, and VA

^c^ Among women who reported breastfeeding < 10 weeks; sites that asked this question included: AL, IA, IN, KY, ME, MI, MO, MT, NC, ND, NE, NH, YC, NY, PR, SD, VA, WA, and WY

^d^ There were no women who identified as Alaska Native or Native Hawaiian within the subset of states that asked additional questions about reasons for not starting or stopping breastfeeding

“Didn’t want to” (47%), “didn’t like it” (27%), and having to take care of children (24%) were the most common reasons women reported for never breastfeeding. American Indian (78%) and Black (51%) women were most likely to report “didn’t want to” as the reason for not breastfeeding, Table 1. Compared to other groups, American Indian women were more likely to report being sick or on medications (29%) or having to go back to work or school (50%, combined) as reasons for not breastfeeding. Over a quarter of Hispanic women (29%) reported trying to breastfeed but found it too difficult.

Among women who stopped breastfeeding before 10 weeks post-partum, the most common reasons included not producing enough milk (57%), baby experiencing difficulty latching (38%), and breastmilk alone not satisfying their baby (38%). Not producing enough milk was a common reason for stopping breastfeeding among all groups (49%-65%). We saw fewer racial/ethnic differences among reasons for stopping breastfeeding, Table 1. About 1 in 5 women who did not breastfeed or breastfed for < 10 weeks reported returning to work or school as a reason for not breastfeeding or stopping; this was more common among American Indian and Black women, Supplemental Fig. 1.

Personal doctor was the most prevalent source of breastfeeding information (77%) followed by nurse/midwife/doula (74%), baby’s doctor (68%), and family/friends (64%), Supplemental Table 2. Support groups (23.3%) and hotline/toll-free numbers (10%) were the least common. A majority of women (73%) reported talking to a lactation/breastfeeding specialist. Almost all women reported receiving breastfeeding information from multiple sources (mean number of sources 3.98; SD 1.7). Overall, 24% of women reported receiving information from a personal doctor, baby’s doctor, family/friends, and nurse/midwife/doula, 10% reported receiving information from a personal doctor, baby’s doctor, and nurse/midwife/doula, and 8% received information from every source except for a hotline, Supplemental Fig. 2.

After adjustment, receiving information from family/friends (aOR = 2.14, 95% CI = 2.01–2.29) or a support group (aOR = 2.02, 95% CI = 1.85–2.19) doubled the likelihood that a woman would breastfeed for ≥ 10 weeks, compared to not receiving information from the source. Information from family/friends (aOR = 1.93, 95% CI = 1.80–2.07) or a support group (aOR = 1.46, 95% CI = 1.33–1.59) increased the odds also of breastfeeding for < 10 weeks, compared to never breastfeeding, Table 2. Information from the baby’s doctor and from a nurse/midwife/doula were also associated with increased breastfeeding; receiving information from a personal doctor or a hotline did not increase breastfeeding initiation or duration, Table 2.

**Table 2:** Prevalence of sources of breastfeeding information, stratified across breastfeeding initiation and duration, and adjusted associations between breastfeeding information from sources and likelihood of breastfeeding < 10 weeks and ≥ 10 weeks (vs. not breastfeeding), weighted to be nationally representative, Pregnancy Risk Assessment Monitoring System Phase 8, 2016–2019. Due to small sample size (*n *= 44 unweighted), Native Hawaiian women were excluded from the analyses

	**Breastfed ≥ 10 weeks**		**Breastfed < 10 weeks**		**Did not Breastfeed (reference)**
	%	aOR (95% CI)^a^	%	aOR (95% CI)^a^	%
**Sources of information**					
Baby’s doctor	61.9	1.43 (1.33, 1.54)	27.4	1.40 (1.29, 1.51)	10.3
Personal doctor	59.1	0.49 (0.45, 0.53)	28.1	0.78 (0.71, 0.85)	12.9
Family/friends	64.9	2.14 (2.01, 2.29)	26.6	1.93 (1.80, 2.07)	8.5
Support group	66.6	2.02 (1.85, 2.19)	25.9	1.46 (1.33, 1.59)	7.5
Hotline	61.1	0.83 (0.74, 0.92)	29.3	0.94 (0.84, 1.06)	9.6
Nurse/midwife/doula	63.4	1.71 (1.60, 1.84)	26.3	1.45 (1.35, 1.57)	10.3

*Abbreviations*: *OR* odds ratio, *CI* confidence interval

^a^ Adjusted for age at birth, education, Kotelchuck index (prenatal care), race/ethnicity, and all other sources of breastfeeding information; reference group = did not breastfeed

### Race/ethnicity-stratified effects of breastfeeding information

**Fig. 1:**
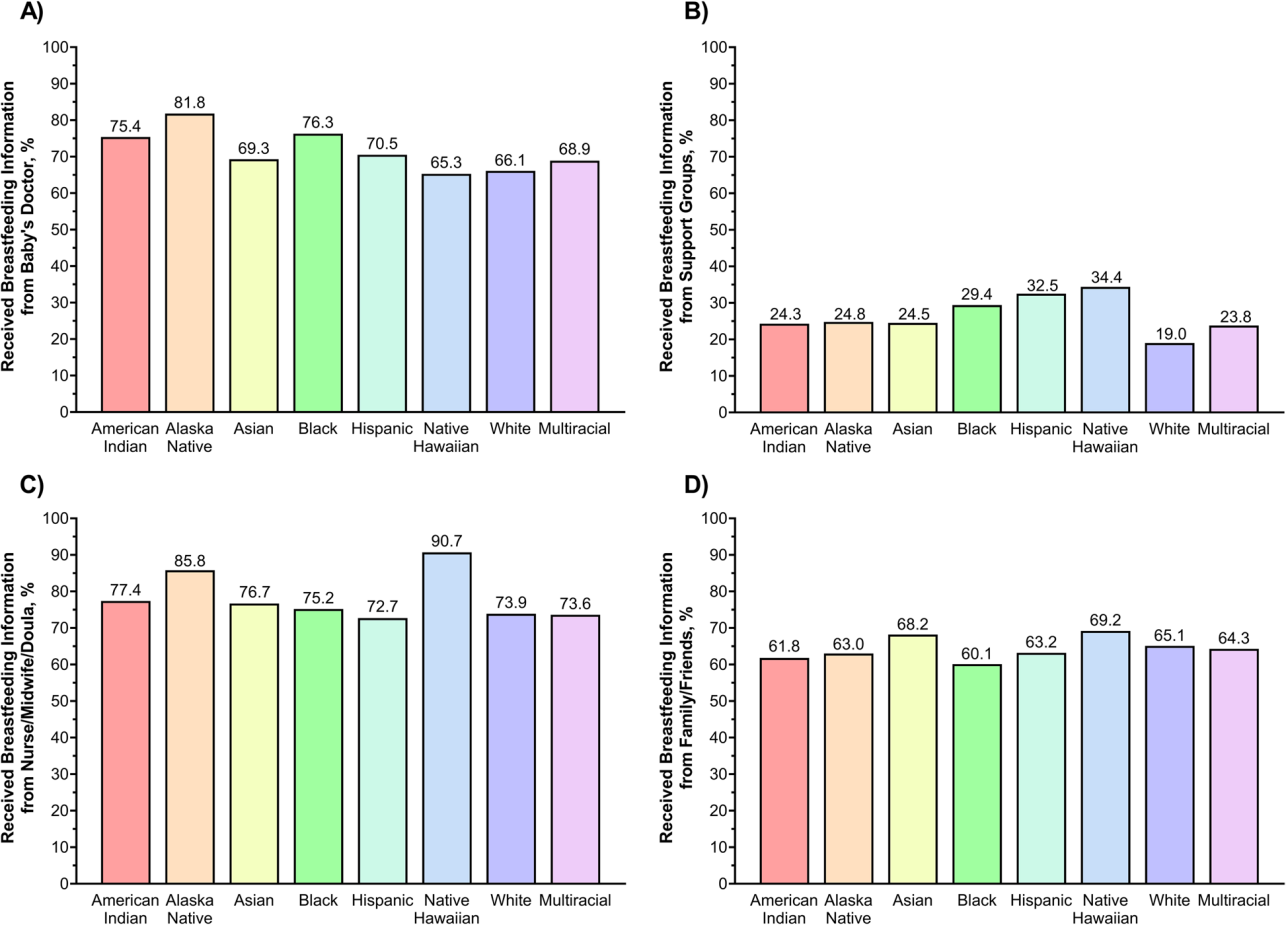
The percentage of women who reported receiving breastfeeding information from **A** baby’s doctor, **B** support groups, **C** nurse/midwife/doula, and **D** family or friends before or after giving birth, stratified by race/ethnicity, weighted to be nationally representative, Pregnancy Risk Assessment Monitoring System Phase 8, 2016–2019

The proportion of women who received breastfeeding information from each source, stratified by race/ethnicity, are presented in Fig. 1 and Supplemental Table 2. Alaska Native (82%), Black (76%), and American Indian (75%) women were more likely to receive breastfeeding information from the baby’s doctor vs. other groups (65–70%), Fig. 1A. Support groups were more commonly used by Native Hawaiian (34%), Hispanic (33%), and Black (29%) women, compared to other racial/ethnic groups (19–25%), Fig. 1B. Almost all Native Hawaiian women (91%) reported receiving information from their nurse/midwife/doula, although rates were also high in the other groups as well (73–86%), Fig. 1C. Fewer racial/ethnic differences were seen in receiving information from family/friends, Fig. 1D.

**Fig. 2:**
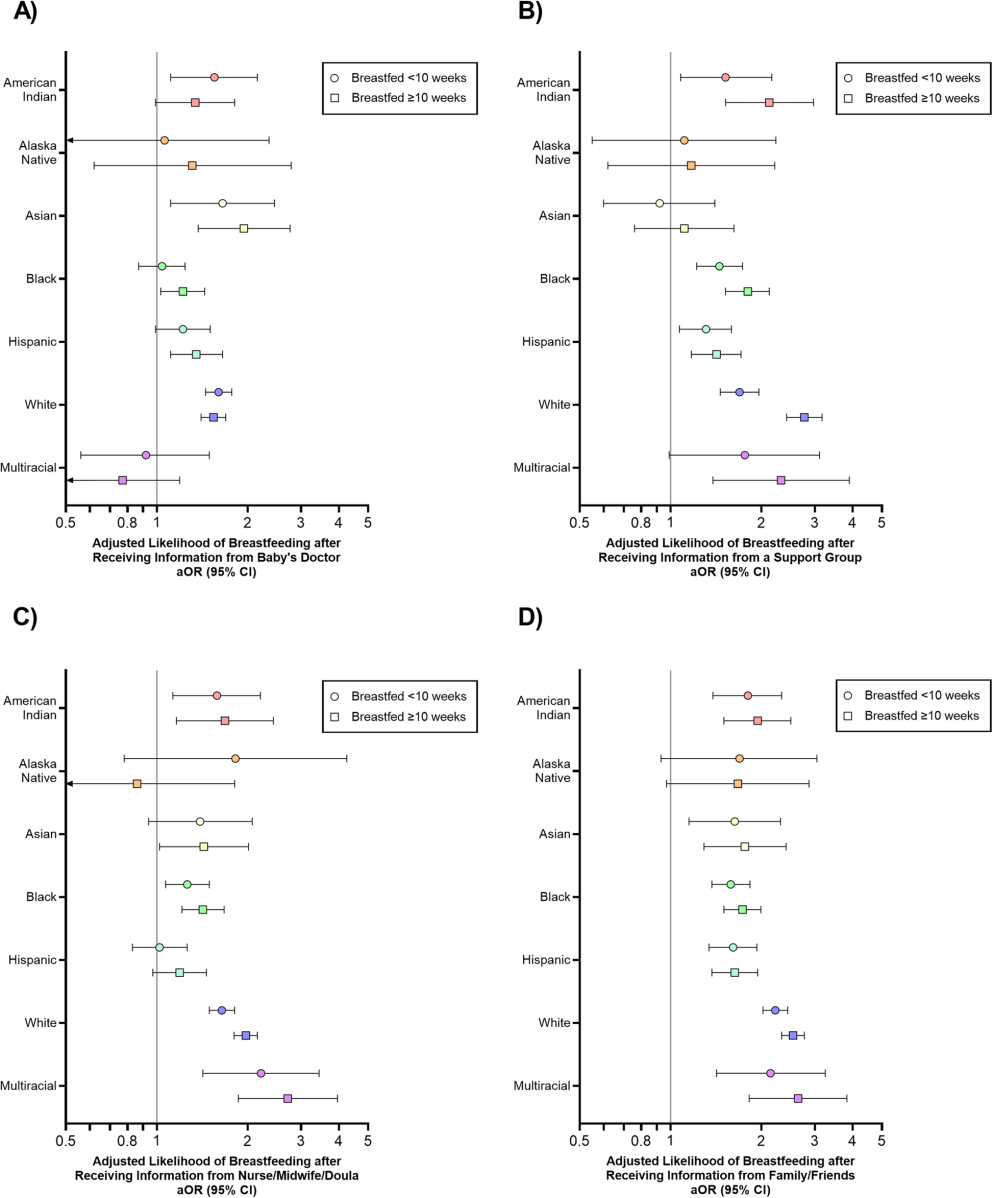
The association between breastfeeding information from **A** baby’s doctor, **B** support groups, **C** nurse/midwife/doula, and **D** family or friends before or after giving birth and breastfeeding duration < 10 weeks and ≥ 10 weeks (vs. not breastfeeding), stratified by race/ethnicity, weighted to be nationally representative, Pregnancy Risk Assessment Monitoring System Phase 8, 2016–2019. Due to small sample size (*n* = 44 unweighted), Native Hawaiian women were excluded from the analyses

Information from the baby’s doctor increased breastfeeding for < 10 weeks among American Indian, Asian, and White women (< 10 weeks: aORs = 1.55–1.76; ≥ 10 weeks: aORs = 1.34–1.60); information from the baby’s doctor also increased the odds of Black and Hispanic women breastfeeding ≥ 10 weeks (aOR = 1.22 and 1.35, respectively), Fig. 2A and Table 3.

**Table 3:** Effect of receiving breastfeeding information from each information source on breastfeeding < 10 weeks and ≥ 10 weeks (compared to not breastfeeding), stratified by race/ethnicity, weighted to be site representative, Pregnancy Risk Assessment Monitoring System Phase 8, 2016–2019

	**Baby’s Doctor**	**Personal Doctor**	**Family/Friends**	**Support Group**	**Hotline**	**Nurse/Midwife/Doula**
	**aOR (95% CI)**^**a**^	**aOR (95% CI)**^**a**^	**aOR (95% CI)**^**a**^	**aOR (95% CI)**^**a**^	**aOR (95% CI)**^**a**^	**aOR (95% CI)**^**a**^
	**Breastfed < 10 weeks**					
**Race/ethnicity**						
American Indian	1.55 (1.11, 2.15)	0.72 (0.51, 1.02)	1.80 (1.38, 2.33)	1.52 (1.08, 2.16)	0.76 (0.48, 1.21)	1.58 (1.13, 2.20)
Alaska Native	1.06 (0.48, 2.35)	1.11 (0.45, 2.73)	1.69 (0.93, 3.05)	1.11 (0.55, 2.23)	1.14 (0.45, 2.91)	1.82 (0.78, 4.25)
Asian	1.65 (1.11, 2.45)	0.74 (0.47, 1.16)	1.63 (1.15, 2.31)	0.92 (0.60, 1.40)	0.82 (0.50, 1.32)	1.39 (0.94, 2.07)
Black	1.04 (0.87, 1.24)	1.05 (0.84, 1.31)	1.58 (1.37, 1.83)	1.45 (1.22, 1.73)	0.97 (0.79, 1.20)	1.26 (1.07, 1.49)
Hispanic	1.22 (0.99, 1.50)	0.81 (0.63, 1.05)	1.61 (1.34, 1.93)	1.31 (1.07, 1.59)	0.99 (0.77, 1.26)	1.02 (0.83, 1.26)
White	1.60 (1.45, 1.77)	0.70 (0.63, 0.79)	2.22 (2.02, 2.44)	1.69 (1.46, 1.96)	1.04 (0.85, 1.26)	1.64 (1.49, 1.81)
Mixed race	0.92 (0.56, 1.49)	1.19 (0.68, 2.08)	2.14 (1.42, 3.25)	1.76 (0.99, 3.11)	1.24 (0.61, 2.54)	2.21 (1.42, 3.44)
	**Breastfed ≥ 10 weeks**					
**Race/ethnicity**						
American Indian	1.34 (0.99, 1.81)	0.59 (0.40, 0.87)	1.94 (1.50, 2.50)	2.12 (1.52, 2.97)	0.74 (0.48, 1.14)	1.68 (1.16, 2.43)
Alaska Native	1.31 (0.62, 2.78)	0.90 (0.40, 2.03)	1.67 (0.97, 2.87)	1.17 (0.62, 2.21)	0.64 (0.27, 1.53)	0.86 (0.41, 1.81)
Asian	1.94 (1.37, 2.76)	0.63 (0.42, 0.94)	1.76 (1.29, 2.41)	1.11 (0.76, 1.62)	0.73 (0.48, 1.12)	1.43 (1.02, 2.01)
Black	1.22 (1.03, 1.44)	0.61 (0.50, 0.74)	1.73 (1.50, 1.99)	1.80 (1.52, 2.12)	0.89 (0.72, 1.10)	1.42 (1.21, 1.67)
Hispanic	1.35 (1.11, 1.65)	0.56 (0.44, 0.72)	1.63 (1.37, 1.94)	1.42 (1.17, 1.71)	1.05 (0.83, 1.32)	1.19 (0.97, 1.46)
White	1.54 (1.40, 1.69)	0.43 (0.39, 0.48)	2.54 (2.33, 2.77)	2.77 (2.42, 3.17)	0.80 (0.66, 0.96)	1.97 (1.80, 2.15)
Mixed race	0.77 (0.50, 1.19)	0.59 (0.37, 0.95)	2.64 (1.82, 3.83)	2.32 (1.38, 3.90)	0.94 (0.47, 1.86)	2.71 (1.86, 3.96)

*Abbreviations*: *OR* odds ratio, *CI* confidence interval

^a^ Adjusted for age at delivery, education, and prenatal care (Kotelchuck index); associations within each racial/ethnic group were modeled separately; reference group = did not breastfeed

Support groups only increased breastfeeding rates among American Indian, Black, Hispanic, White, and multiracial women (< 10 weeks: aORs = 1.31–1.76; ≥ 10 weeks: aORs = 1.42–2.77) and had no significant effect among Alaska Native or Asian women, Fig. 2B and Table 3. Information from nurse/midwife/doula increased breastfeeding rates among American Indian, Black, White, and multiracial women (< 10 weeks: aORs = 1.26–2.21; ≥ 10 weeks: aORs = 1.42–2.71), Fig. 2C. Information from family/friends was consistently associated with increased reporting of any breastfeeding and breastfeeding duration across all racial/ethnic groups (< 10 weeks: aORs = 1.58–2.14; ≥ 10 weeks: aORs = 1.63–2.64), Fig. 2D and Table 3. Overall, receiving breastfeeding information from support groups, nurse/midwife/doulas, the baby’s doctor, and family/friends substantially benefitted American Indian, Asian, White, and multiracial women, with weaker associations among Alaska Native, Black, and Hispanic women.

When Hispanic women were stratified by English- and Spanish-speaking, receiving information from family/friends had a greater association with breastfeeding among English-speaking Hispanic women (< 10 weeks: aOR = 1.81, 95% CI 1.44–2.28; ≥ 10 weeks: aOR = 2.02, 95% CI = 1.62–2.52) compared to their Spanish-speaking counterparts (< 10 weeks: aOR = 1.33, 95% CI 1.00–1.77; ≥ 10 weeks: aOR = 1.25, 95% CI = 0.96–1.63), Supplemental Table 3. Despite a greater percentage of Spanish-speaking Hispanic women obtaining information from a support group compared with their English-speaking counterparts (39.5% vs. 27.4%), support groups improved breastfeeding among English-speaking Hispanic women (< 10 weeks: aOR = 1.39, 95% CI 1.05–1.83; ≥ 10 weeks: aOR = 1.63, 95% CI = 1.25–2.12) with no statistically significant impact among their Spanish-speaking peers (< 10 weeks: aOR = 1.16, 95% CI 0.86–1.57; ≥ 10 weeks: aOR = 1.11, 95% CI = 0.83–1.47), Supplemental Table 3. Receiving breastfeeding information from the baby’s doctor was also only associated with increased breastfeeding among English-speaking Hispanic women (< 10 weeks: aOR = 1.42, 95% CI = 1.10–1.84; ≥ 10 weeks aOR = 1.65, 95% CI = 1.29, 2.11), Supplemental Table 3.

When lactation specialist was included in the models, results were relatively consistent, although baby’s doctor was no longer significantly associated with increased breastfeeding, Supplemental Table 4. Receipt of information from lactation specialists was strongly associated with increased breastfeeding (< 10 weeks: aOR = 5.18, 95% CI = 4.83–5.56; ≥ 10 weeks aOR = 5.25, 95% CI = 4.86–5.67). The association between lactation specialists and breastfeeding was statistically significant among all racial/ethnic groups but was stronger among White women (< 10 weeks: aOR = 7.39, 95% CI = 6.65–8.22; ≥ 10 weeks aOR = 7.17, 95% CI = 6.52–7.87), compared to other racial-ethnic groups (< 10 weeks: aORs = 2.44–4.83; ≥ 10 weeks aOR = 2.79–4.30), Supplemental Table 5.

## Discussion

In an analysis of nationally representative surveillance data, we found that receiving breastfeeding information during pregnancy or shortly after delivery was common, with a personal doctor, nurse/midwife/doula, and baby’s doctor being the most common sources. While family/friends, support groups, baby’s doctor, and nurse/midwife/doula were consistently associated with increased breastfeeding rates, the effects of these sources were smaller among Alaska Native, Black, and Hispanic women, compared to White women. Receiving information from a personal doctor or from breastfeeding hotlines did not increase breastfeeding initiation/duration among any groups. Thus, the impact of breastfeeding information varied by source and across race/ethnicity.

Similar to other studies which have found that support groups have multiple benefits including increasing breastfeeding, [17, 18] we found that information from a support group was strongly and consistently associated with breastfeeding initiation/duration among American Indian, Black, Hispanic (English-speaking only), White, and multiracial women; however, the association was smaller among English-speaking Hispanic and Black women, and absent among Alaska Native, Asian, or Spanish-speaking Hispanic women. Our study indicated that racial/ethnic disparities exist related to effect of breastfeeding information on breastfeeding initiation/duration, such that this information tends to be less effective for certain ethnic populations. These differences may be due to the dearth of culturally tailored support groups that meet the needs of marginalized populations, and/or deliver information in participant’s native languages. Most randomized trials of breastfeeding support groups have been conducted among non-Hispanic White women, and culturally tailored interventions for racial/ethnic minority women have been generally graded as lower-quality in a systematic review [9]. Access to breastfeeding support groups for diverse racial/ethnic populations may also be an issue. At least one study has found that access to Special Supplemental Nutrition Program for Women, Infants, and Children breastfeeding support services, a federal assistance program for healthcare and nutrition for low-income populations, was most limited in predominantly Black communities [19]. More efforts are needed to improve access to culturally tailored breastfeeding supportive services, especially in under-resourced communities.

Consistent with prior studies showing that people rely heavily on their social support networks for guidance and advice for breastfeeding, [20] in our study, family and friends were also an important source for increasing breastfeeding initiation/duration among all racial/ethnic groups except Spanish-speaking Hispanic women. Despite the importance of friends and family in breastfeeding, Black and American Indian women were less likely to report receiving breastfeeding information from their family/friends compared to other racial/ethnic groups. Prior studies suggest that lack of familial support is a major barrier to breastfeeding [20]. Among inner city African American women, research has shown that they were less likely to witness an African American woman breastfeeding within their community and were less likely to receive supportive breastfeeding advice from family and friends [21]. In our study, Black and American Indian women were also most likely to report not wanting to breastfeed or not liking breastfeeding. This may be due to intergenerational and historical trauma of White supremacy (e.g., oppression, gendered dehumanization of enslavement, wet nursing, genocide, cultural erasure, and forced removal from ancestral lands) among American Indian and African American women and breastfeeding persons [22, 23]. The decision to breastfeed among African American women remains deeply attached to the generational trauma of wet nurses during slavery where African American women were forced to breastfeed White children at the expense of their own, perpetuating the stereotype of African American infants being needy, sicker, and less well-behaved [23]. A promising approach among American Indian women is employing grandmothers as advocates to strengthen cultural ties to traditional breastfeeding practices, which has been shown to significantly increase breastfeeding rates [24].

Social media and online resources are also important sources to consider. The use of social media for breastfeeding support groups is associated with longer breastfeeding duration through the sharing of knowledge that can increase positive breastfeeding experiences, social connections, and a sense of belonging and breastfeeding self-efficacy [25]. Technology and social media can be leveraged to provide real-time help, such as peer support provided via texting, which was found to increase rates among Hispanic women [26]. However, social media can also spread misinformation and unmoderated support groups can contain inaccurate information. Although not captured directly in this study, misinformation could account in part for the proportion of women reporting “other” as a reason for not initiating or stopping breastfeeding.

Our findings have implications for public health, clinical, and policy efforts to decrease racial/ethnic disparities in breastfeeding. The Centers for Disease Control and Prevention has previously outlined the need to increase peer support programs and inclusion of relatives (e.g., spouses, grandmothers) into breastfeeding programming [27]. Programs that leverage family and friends as a source of breastfeeding information and culturally and linguistically appropriate peer-delivered community-based interventions have been shown to significantly reduce racial/ethnic disparities in breastfeeding [9, 12, 24, 28]. These findings point to the potential benefits of family and friend centered interventions, especially, among Spanish-speaking Hispanic women. The high rates of receipt of information from nurses, midwives and doulas and high rates of longer-term breastfeeding among Native Hawaiian women in particular could reflect such contextually embedded programming and needs to be better understood. For Asian women, further understanding of the heterogeneity across sub-populations might also inform cultural tailoring of breastfeeding interventions and increase their effectiveness. Further disaggregation of Asian women was not possible in our analysis due to small sample sizes, but further analysis is needed to understand the nuances of breast feeding behaviors within this group.

Although information from a personal doctor was the most prevalent source of breastfeeding information, it was not associated with increased breastfeeding initiation/duration overall or among any racial/ethnic group. By contrast, information from the baby’s doctor was slightly less common (68.6% vs. 77.2%), and it was associated with increased breastfeeding rates among almost all racial/ethnic groups. Notably, receiving information from the baby’s doctor was associated with longer breastfeeding among English-speaking Hispanic women but not among those who were Spanish speaking, possibly due to the need for and lack of language concordant physicians and trained interpreters to assist in the provision of breastfeeding information during visits. Strong encouragement from language concordant physicians could be effective among Spanish-speaking Hispanic women who tend to defer to physicians.

The limited impact of breastfeeding information from a personal doctor may be due to time constraints during appointments, leading to prioritization of other health topics, [29] which limits the quantity and quality of breastfeeding information imparted and received. The limited impact of personal physicians might also be due to barriers in accessing post-partum care and missing postpartum appointments due to work time constraints [30]. Furthermore, our findings that the main reasons for stopping breastfeeding were not producing enough milk, difficulty latching, and not enough breastmilk, indicate that early support is critical [31]. Increasing the availability of resources and remote surveillance, and bundling of services, e.g., referring women to toll-free hotlines, support groups, and lactation specialists, could result in greater impact. For example, toll-free hotlines were underutilized (only 10% of women reporting accessing these) and could provide real-time support (especially if accessible in multiple languages), supplementing other sources of information.

Of note, only family/friends appeared to be consistently associated with breastfeeding among Alaska Native women, suggesting that resources provided through clinicians and support groups are not adequately meeting their needs. Since all Alaska Native women in our sample resided in Alaska, information sources and public health programming in Alaska may need to be made more easily accessible and culturally tailored for Alaska Native women.

Improving access to and quality of breastfeeding information and resources will not fully address breastfeeding disparities or maximize breastfeeding rates in the U.S. In our study, among women who did not breastfeed, one-quarter of Black women and half of American Indian women reported not initiating breastfeeding because they had to go back to work or school. Among those who breastfed < 10 weeks, 28% of Black and 20% of American Indian women reporting stopping because they had to go back to work or school. Overall, almost 1 in 5 women who did not breastfeed for ≥ 10 weeks reported returning to the workforce/school as a reason for stopping/not initiating breastfeeding.

Lack of access to space, time, and resources for breastfeeding or pumping at work or school need to be addressed. Consistent national workplace protections could help increase breastfeeding duration [20]. Intention to work full-time as well as shorter work leaves are both associated with lower rates of initiation and shorter breastfeeding duration [20, 32]. The U.S. federal Family and Medical Leave Act (FMLA), a labor law requiring employers to provide employees with job-protected unpaid leave, only offers leave for up to 12 weeks (much shorter than the recommended 6 months of breastfeeding), and 40% of the workforce is ineligible for FMLA, leaving many post-partum people from low-income and racial/ethnic minority groups unable to take work leave [33]. Support programs and policies which increase access to paid leave, school and workplace accommodations (e.g., providing private and comfortable lactation sites) are crucial to meet the Healthy People 2030 goal of 42.4% of infants exclusively breastfed for the first six months, [34] and may provide the best avenue for reducing breastfeeding disparities [33].

This study has limitations. First, given the nature of the PRAMS dataset and available questions, we were unable to measure or account for other important covariates such as 1) quantity, quality, and content of breastfeeding information, including if the woman gave birth at a hospital with breastfeeding initiatives, 2) access to paid leave and workplace protections for pumping/breastfeeding and other resources needed to promote breastfeeding after returning to work, or 3) participants’ views on breastfeeding (e.g., support, time commitment, exhaustion, experiences of discrimination). Second, since PRAMS surveys women within the first few post-partum months, we were unable to assess the proportion of women who met the recommendation to breastfeed for the first 6 months and its association with specific types of breastfeeding information sources,overall or among specific racial/ethnic groups. Although one of the strengths of this study was the ability to stratify individuals into the major racial/ethnic groups, they are still large and heterogeneous, e.g., Asian women. It is likely that varied cultural practices and values surrounding breastfeeding exist within these groups. For example, there are significant differences in breastfeeding initiation between Black immigrants and African Americans [21, 35]. Future studies should sample to allow for further disaggregation of these populations. Third, recall bias may be present as all PRAMS data on breastfeeding, including breastfeeding information sources are self-reported. However, we believe this bias would be minimal because the surveys were conducted within the post-partum period. Finally, due to the small sample size of Native Hawaiian women in PRAMS, we were unable to assess the impact of breastfeeding information sources on breastfeeding rates in this group. Future studies are needed to better understand breastfeeding practices in this population.

## Conclusions

Using multi-state, nationally representative surveillance data from 2016–2019, we found that breastfeeding disparities continue to persist among diverse racial/ethnic groups despite ongoing public health efforts. Receiving breastfeeding information from family, friends, or a support group were consistently associated with improved breastfeeding rates; however, these resources had smaller or no impact among Alaska Native, Black, and Hispanic women. Receiving information from the baby’s doctor also had limited benefit among racial/ethnic minorities, and receipt of breastfeeding information from a personal doctor had no impact on breastfeeding initiation/duration. Public health interventions should consider enhancing clinician information with other breastfeeding information and support services, including early surveillance and assistance. Improving access to and acceptability of breastfeeding among communities through linguistically and culturally appropriate efforts continues to be a public health challenge. Finally, while individual resources may improve breastfeeding rates among racial/ethnic minorities, paid leave and protections for pregnant and breastfeeding individuals in the workplace or schools are needed to address breastfeeding disparities in the U.S. and improve health equity among marginalized populations.

## Supplementary Information

**Additional file 1: Supplemental Table 1.** Demographics of women, stratified by race/ethnicity, weighted to be site representative, Pregnancy Risk Assessment Monitoring System Phase 8, 2016-2019. **Supplemental Figure 1.** Women having to go back to work or school as reason for not breastfeeding or stopping breastfeeding (<10 weeks), stratified by race/ethnicity, weighted to be site representative, Pregnancy Risk Assessment Monitoring System Phase 8, 2016-2019. **Supplemental Table 2.** Percentage of women who received breastfeeding information from each source, stratified by race/ethnicity, weighted to be site representative, Pregnancy Risk Assessment Monitoring System Phase 8, 2016-2019. **Supplemental Figure 2.** Frequency of women receiving information from various combinations of breastfeeding information sources, sorted by most to least prevalent, weighted to be site representative, Pregnancy Risk Assessment Monitoring System Phase 8, 2016-2019. Black dots indicate that breastfeeding source was part of the combination (e.g., the first bar represents the number of women who received information from family/friends, baby’s doctor, nurse/midwife/doula, and their personal doctor). The bars next to the information sources under the x-axis represent the total number of women who received information from that source. **Supplemental Table 3.** Effect of receiving breastfeeding information from each information source on any breastfeeding and breastfeeding duration, stratified by English and Spanish speaking Hispanic women, weighted to be site representative, Pregnancy Risk Assessment Monitoring System Phase 8, 2016-2019. **Supplemental Table 4.** Prevalence of sources of breastfeeding information, stratified across any breastfeeding and breastfeeding duration, and adjusted associations between breastfeeding information from sources and likelihood of breastfeeding <10 weeks and ≥10 weeks (vs. not breastfeeding), weighted to be nationally representative, Pregnancy Risk Assessment Monitoring System Phase 8, 2016-2019. Due to small sample size (n=44 unweighted), Native Hawaiian women were excluded from the analyses. **Supplemental Table 5.** Effect of receiving breastfeeding information from a lactation specialist on breastfeeding <10 weeks and ≥10 weeks (compared to not breastfeeding), stratified by race/ethnicity, weighted to be site representative, Pregnancy Risk Assessment Monitoring System Phase 8, 2016-2019.

## Data Availability

The datasets generated and/or analyzed during the current study used data from the Pregnancy Risk Assessment Monitoring System, a surveillance project of the CDC and state, territorial, or tribal health departments. Data is available upon request from the CDC by submitting a proposal to PRAMSProposals@cdc.gov. Approved proposals will then be forwarded to the PRAMS sites for review and datasets are sent out approximately 6 weeks after the date of initial review. For more information please visit, [https://www.cdc.gov/prams/prams-data/researchers.htm].

## Abbreviations

U.S: United States
PRAMS: Pregnancy Risk Assessment Monitoring System
SD: Standard deviation
CI: Confidence interval
aOR: Adjusted odds ratio
FMLA: Family and Medical Leave Act
