# Supplementary material for: Race/ethnicity-specific associations between breastfeeding information source and breastfeeding rates among U.S. women
Source: BMC Public Health. 2023 Mar 17;23:520. doi: 10.1186/s12889-023-15447-8 (PMC10024358; doi:10.1186/s12889-023-15447-8)
Supplement: Supplementary file 1 — Additional file 1: Supplemental Table 1. Demographics of women, stratified by race/ethnicity, weighted to be site representative, Pregnancy Risk Assessment Monitoring System Phase 8, 2016-2019. Supplemental Figure 1. Women having to go back to work or school as reason for not breastfeeding or stopping breastfeeding (<10 weeks), stratified by race/ethnicity, weighted to be site representative, Pregnancy Risk Assessment Monitoring System Phase 8, 2016-2019. Supplemental Table 2. Percentage of women who received breastfeeding information from each source, stratified by race/ethnicity, weighted to be site representative, Pregnancy Risk Assessment Monitoring System Phase 8, 2016-2019. Supplemental Figure 2. Frequency of women receiving information from various combinations of breastfeeding information sources, sorted by most to least prevalent, weighted to be site representative, Pregnancy Risk Assessment Monitoring System Phase 8, 2016-2019. Black dots indicate that breastfeeding source was part of the combination (e.g., the first bar represents the number of women who received information from family/friends, baby’s doctor, nurse/midwife/doula, and their personal doctor). The bars next to the information sources under the x-axis represent the total number of women who received information from that source. Supplemental Table 3. Effect of receiving breastfeeding information from each information source on any breastfeeding and breastfeeding duration, stratified by English and Spanish speaking Hispanic women, weighted to be site representative, Pregnancy Risk Assessment Monitoring System Phase 8, 2016-2019. Supplemental Table 4. Prevalence of sources of breastfeeding information, stratified across any breastfeeding and breastfeeding duration, and adjusted associations between breastfeeding information from sources and likelihood of breastfeeding <10 weeks and ≥10 weeks (vs. not breastfeeding), weighted to be nationally representative, Pregnancy Risk Assessment Monitoring [file 12889_2023_15447_MOESM1_ESM.docx]

Supplemental Table 1. Demographics of women, stratified by race/ethnicity, weighted to be site representative, Pregnancy Risk Assessment Monitoring System Phase 8, 2016-2019.

|  | **American**  **Indian** | | **Alaska Native** | | **Asian** | | **Black** | | **Hispanic** | | **Mixed Race** | | **Native Hawaiian** | | **White** | |
| --- | --- | --- | --- | --- | --- | --- | --- | --- | --- | --- | --- | --- | --- | --- | --- | --- |
| **Total, N** | 37,011 | | 6,069 | | 316,708 | | 850,481 | | 1,046,558 | | 139,794 | | 973 | | 3,547,426 | |
| **Maternal age, n (%)** |  |  |  |  |  |  |  |  |  |  |  |  |  |  |  |  |
| 18-19 | 2,542 | (6.9) | 410 | (6.8) | 1,882 | (0.6) | 38,997 | (4.6) | 58,164 | (5.6) | 7,319 | (5.2) | 26 | (2.7) | 83,988 | (2.4) |
| 20-24 | 10,045 | (27.1) | 1,685 | (27.8) | 23,878 | (7.5) | 215,469 | (25.3) | 269,596 | (25.8) | 36,701 | (26.3) | 64 | (6.6) | 570,193 | (16.1) |
| 25-29 | 12,461 | (33.7) | 2,194 | (36.2) | 87,679 | (27.7) | 267,285 | (31.4) | 298,875 | (28.6) | 41,938 | (30.0) | 285 | (29.3) | 1,062,263 | (29.9) |
| 30-34 | 7,964 | (21.5) | 1,183 | (19.5) | 124,400 | (39.3) | 198,401 | (23.3) | 250,596 | (23.9) | 32,574 | (23.3) | 308 | (31.6) | 1,173,714 | (33.1) |
| 35-39 | 3,062 | (8.3) | 518 | (8.5) | 64,337 | (20.3) | 103,775 | (12.2) | 134,088 | (12.8) | 16,991 | (12.2) | 161 | (16.6) | 550,643 | (15.5) |
| 40+ | 936 | (2.5) | 79 | (1.3) | 14,531 | (4.6) | 26,554 | (3.1) | 35,239 | (3.4) | 4,271 | (3.1) | 128 | (13.2) | 106,625 | (3.0) |
| **Maternal education, n (%)** |  |  |  |  |  |  |  |  |  |  |  |  |  |  |  |  |
| Less than high school degree | 6,893 | (18.6) | 1,207 | (19.9) | 20,788 | (6.6) | 86,901 | (10.2) | 267,441 | (25.6) | 15,308 | (11.0) | 31 | (3.2) | 203,318 | (5.7) |
| High school degree | 13,644 | (37.0) | 3,162 | (52.5) | 39,716 | (12.6) | 304,840 | (36.1) | 329,769 | (31.8) | 36,247 | (26.1) | 476 | (49.0) | 714,795 | (20.2) |
| Some college | 12,647 | (34.3) | 1,411 | (23.5) | 54,217 | (17.2) | 295,174 | (35.0) | 271,859 | (26.2) | 49,613 | (35.7) | 291 | (29.9) | 958,803 | (27.1) |
| College degree or higher | 3,670 | (10.0) | 237 | (3.9) | 200,541 | (63.6) | 157,453 | (18.6) | 166,852 | (16.1) | 37,905 | (27.3) | 175 | (18.0) | 1,655,497 | (46.9) |
| **Married, n (%)** | 12,712 | (34.4) | 1,968 | (32.5) | 275,774 | (87.1) | 248,542 | (29.2) | 507,770 | (48.6) | 70,514 | (50.4) | 534 | (54.9) | 2,627,623 | (74.1) |
| **Insurance, n (%)** |  |  |  |  |  |  |  |  |  |  |  |  |  |  |  |  |
| Private insurance | 8,001 | (21.8) | 752 | (12.7) | 212,476 | (67.4) | 258,346 | (30.5) | 301,322 | (29.0) | 60,480 | (43.5) | 361 | (37.1) | 2,348,872 | (66.8) |
| Medicaid | 24,374 | (66.4) | 4,307 | (72.8) | 91,671 | (29.1) | 553,446 | (65.4) | 616,748 | (59.3) | 68,505 | (49.3) | 587 | (60.3) | 971,306 | (27.6) |
| Other government insurance | 2,920 | (8.0) | 815 | (13.8) | 5,323 | (1.7) | 12,694 | (1.5) | 19,339 | (1.9) | 6,405 | (4.6) | 23 | (2.3) | 73,866 | (2.1) |
| Uninsured/self-pay | 511 | (1.4) | 32 | (0.5) | 3,688 | (1.2) | 10,770 | (1.3) | 74,307 | (7.1) | 2,039 | (1.5) | 2 | (0.2) | 83,752 | (2.4) |
| Other | 902 | (2.5) | 7 | (0.1) | 2,184 | (0.7) | 11,152 | (1.3) | 28,055 | (2.7) | 1,588 | (1.1) |  |  | 39,249 | (1.1) |
| **Total income^a^, n (%)** |  |  |  |  |  |  |  |  |  |  |  |  |  |  |  |  |
| $0-$20,000 | 17,646 | (51.1) | 2,674 | (49.0) | 51,303 | (17.7) | 374,719 | (49.6) | 391,841 | (43.5) | 42,726 | (32.6) | 125 | (17.9) | 562,678 | (16.6) |
| $20,001-$40,000 | 8,308 | (24.1) | 1,610 | (29.5) | 46,258 | (16.0) | 198,353 | (26.2) | 259,303 | (28.8) | 31,076 | (23.7) | 126 | (18.0) | 581,502 | (17.2) |
| $40,001-$60,000 | 4,073 | (11.8) | 509 | (9.3) | 31,846 | (11.0) | 76,998 | (10.2) | 106,119 | (11.8) | 18,151 | (13.8) | 229 | (32.7) | 486,420 | (14.4) |
| $60,001-$85,000 | 2,445 | (7.1) | 349 | (6.4) | 34,660 | (12.0) | 43,192 | (5.7) | 52,071 | (5.8) | 13,100 | (10.0) | 2 | (0.2) | 481,073 | (14.2) |
| ≥$85,001 | 2,050 | (5.9) | 317 | (5.8) | 125,164 | (43.3) | 62,574 | (8.3) | 90,923 | (10.1) | 26,198 | (20.0) | 217 | (31.1) | 1,276,971 | (37.7) |
| **Census division, n (%)** |  |  |  |  |  |  |  |  |  |  |  |  |  |  |  |  |
| New England | 554 | (1.9) | . | . | 27,942 | (9.6) | 35,475 | (4.3) | 73,102 | (8.2) | 9,592 | (7.1) | 27 | (2.9) | 258,066 | (7.6) |
| Middle Atlantic | 403 | (1.4) | . | . | 79,140 | (27.3) | 130,697 | (16.0) | 167,258 | (18.8) | 16,154 | (12.0) | 272 | (29.4) | 551,074 | (16.2) |
| East North Central | 3,213 | (10.8) | . | . | 41,859 | (14.4) | 143,846 | (17.6) | 117,215 | (13.2) | 19,912 | (14.8) | . | . | 624,484 | (18.4) |
| West North Central | 7,450 | (25.0) | . | . | 18,909 | (6.5) | 53,630 | (6.6) | 51,249 | (5.8) | 20,648 | (15.4) | 9 | (1.0) | 468,739 | (13.8) |
| South Atlantic | 1,397 | (4.7) | . | . | 45,009 | (15.5) | 238,890 | (29.3) | 126,902 | (14.3) | 12,888 | (9.6) | 27 | (2.9) | 494,269 | (14.5) |
| East South Central | 598 | (2.0) | . | . | 3,823 | (1.3) | 65,807 | (8.1) | 17,402 | (2.0) | 4,681 | (3.5) | . | . | 190,065 | (5.6) |
| West South Central | 8,598 | (28.8) | . | . | 20,892 | (7.2) | 125,178 | (15.3) | 185,968 | (20.9) | 12,217 | (9.1) | . | . | 312,609 | (9.2) |
| Mountain | 3,500 | (11.7) | . | . | 11,931 | (4.1) | 9,306 | (1.1) | 86,640 | (9.7) | 10,383 | (7.7) | 101 | (10.9) | 285,387 | (8.4) |
| Pacific | 4,091 | (13.7) | 6,069 | (100.0) | 40,320 | (13.9) | 13,283 | (1.6) | 64,158 | (7.2) | 27,814 | (20.7) | 490 | (53.0) | 216,415 | (6.4) |
| **Urbanicity, n (%)** |  |  |  |  |  |  |  |  |  |  |  |  |  |  |  |  |
| Urban | 11,317 | (35.9) | 1,811 | (29.8) | 182,016 | (93.3) | 426,536 | (88.4) | 541,214 | (82.3) | 72,649 | (75.4) | 378 | (59.8) | 1,431,869 | (69.4) |
| Rural | 20,222 | (64.1) | 4,258 | (70.2) | 12,977 | (6.7) | 55,759 | (11.6) | 116,451 | (17.7) | 23,728 | (24.6) | 255 | (40.2) | 631,936 | (30.6) |
| ^a^ Total income in the 12 months prior | | | | | | | | | | | | | | | | |
| ^b^ Method of payment on birth certificate | | | | | | | | | | | | | | | | |

Supplemental Figure 1. Women having to go back to work or school as reason for not breastfeeding or stopping breastfeeding (<10 weeks), stratified by race/ethnicity, weighted to be site representative, Pregnancy Risk Assessment Monitoring System Phase 8, 2016-2019.


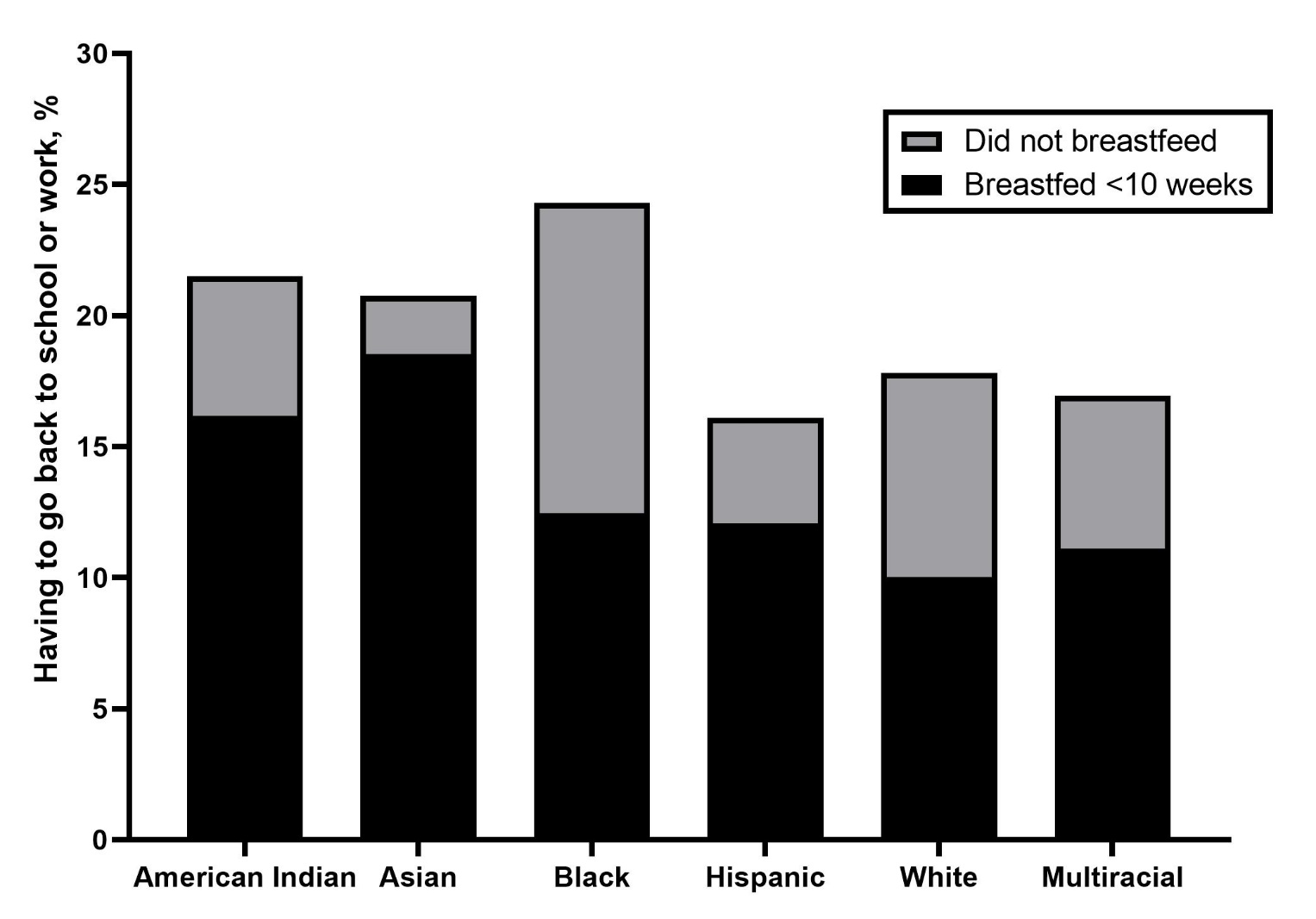


Supplemental Table 2. Percentage of women who received breastfeeding information from each source, stratified by race/ethnicity, weighted to be site representative, Pregnancy Risk Assessment Monitoring System Phase 8, 2016-2019.

|  | **Baby’s**  **Doctor** | **Personal**  **Doctor** | **Family/**  **Friends** | **Support Group** | **Hotline** | **Nurse/**  **Midwife/Doula** | **Lactation**  **Specialist** |
| --- | --- | --- | --- | --- | --- | --- | --- |
|  | **%** | **%** | **%** | **%** | **%** | **%** | **%** |
| **Overall** | 68.6 | 77.2 | 64.2 | 23.3 | 10.6 | 74.1 | 73.9 |
| **Race/ethnicity** |  |  |  |  |  |  |  |
| American Indian | 75.4 | 80.3 | 61.8 | 24.3 | 11.4 | 77.4 | 71.1 |
| Alaska Native | 81.8 | 84.9 | 63.0 | 24.8 | 9.7 | 85.8 | 69.6 |
| Asian | 69.3 | 75.0 | 68.2 | 24.5 | 14.0 | 76.7 | 73.7 |
| Black | 76.3 | 85.5 | 60.1 | 29.4 | 16.1 | 75.2 | 72.4 |
| Hispanic | 70.5 | 78.2 | 63.2 | 32.5 | 16.0 | 72.7 | 74.2 |
| English-speaking | 71.2 | 79.7 | 66.7 | 27.4 | 12.6 | 73.6 | 76.2 |
| Spanish-speaking | 69.5 | 76.1 | 58.4 | 39.5 | 20.7 | 71.5 | 71.6 |
| Native Hawaiian | 65.3 | 65.4 | 69.2 | 34.4 | 11.1 | 90.7 | 54.3 |
| White | 66.1 | 75.0 | 65.1 | 19.0 | 7.4 | 73.9 | 74.2 |
| Mixed race | 68.9 | 79.5 | 64.3 | 23.8 | 9.5 | 73.6 | 75.9 |


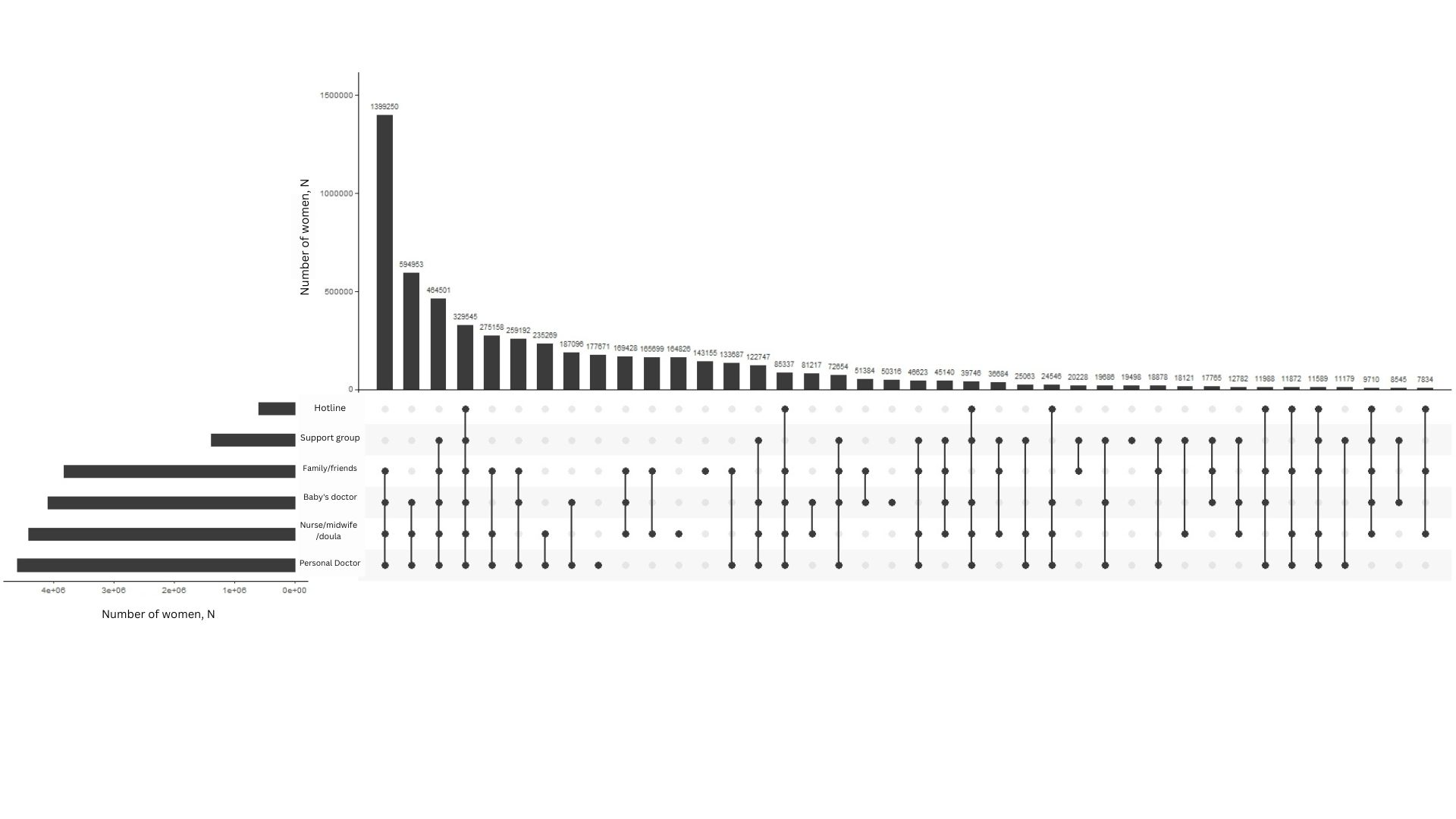
Supplemental Figure 2. Frequency of women receiving information from various combinations of breastfeeding information sources, sorted by most to least prevalent, weighted to be site representative, Pregnancy Risk Assessment Monitoring System Phase 8, 2016-2019. Black dots indicate that breastfeeding source was part of the combination (e.g., the first bar represents the number of women who received information from family/friends, baby’s doctor, nurse/midwife/doula, and their personal doctor). The bars next to the information sources under the x-axis represent the total number of women who received information from that source.

Supplemental Table 3. Effect of receiving breastfeeding information from each information source on any breastfeeding and breastfeeding duration, stratified by English and Spanish speaking Hispanic women, weighted to be site representative, Pregnancy Risk Assessment Monitoring System Phase 8, 2016-2019.

|  | **English Speaking** | **Spanish Speaking** |
| --- | --- | --- |
|  | **aOR (95% CI)^a^** | **aOR (95% CI)^a^** |
|  | **Breastfed <10 weeks** | |
| **Sources of information** |  |  |
| Baby’s doctor | 1.42 (1.10, 1.84) | 0.95 (0.67, 1.33) |
| Personal doctor | 0.60 (0.43, 0.84) | 1.34 (0.90, 2.00) |
| Family/friends | 1.81 (1.44, 2.28) | 1.33 (1.00, 1.77) |
| Support group | 1.39 (1.05, 1.83) | 1.16 (0.86, 1.57) |
| Hotline | 0.92 (0.65, 1.30) | 1.03 (0.73, 1.37) |
| Nurse/midwife/doula | 1.17 (0.90, 1.52) | 0.78 (0.54, 1.11) |
|  | **Breastfed** ≥**10 weeks** | |
| **Sources of information** |  |  |
| Baby’s doctor | 1.65 (1.29, 2.11) | 1.00 (0.73, 1.37) |
| Personal doctor | 0.40 (0.29, 0.55) | 0.99 (0.68, 1.44) |
| Family/friends | 2.02 (1.62, 2.52) | 1.25 (0.96, 1.63) |
| Support group | 1.63 (1.25, 2.12) | 1.11 (0.83, 1.47) |
| Hotline | 0.88 (0.63, 1.23) | 1.18 (0.85, 1.62) |
| Nurse/midwife/doula | 1.19 (0.92, 1.53) | 1.09 (0.78, 1.54) |
| Abbreviations: OR, odds ratio; CI, confidence interval  ^a^ Adjusted for age at birth, education, Kotelchuck index (prenatal care), and all other sources of breastfeeding information; reference group=did not breastfeed | | |

Supplemental Table 4. Prevalence of sources of breastfeeding information, stratified across any breastfeeding and breastfeeding duration, and adjusted associations between breastfeeding information from sources and likelihood of breastfeeding <10 weeks and ≥10 weeks (vs. not breastfeeding), weighted to be nationally representative, Pregnancy Risk Assessment Monitoring System Phase 8, 2016-2019. Due to small sample size (n=44 unweighted), Native Hawaiian women were excluded from the analyses.

|  | **Breastfed ≥10 weeks** | |  | **Breastfed <10 weeks** | |
| --- | --- | --- | --- | --- | --- |
|  | % | aOR (95% CI)^a^ |  | % | aOR (95% CI)^a^ |
| **Sources of information** |  |  |  |  |  |
| Baby’s doctor | 61.9 | 1.06 (0.98, 1.14) |  | 27.4 | 1.03 (0.95, 1.12) |
| Personal doctor | 59.1 | 0.47 (0.43, 0.51) |  | 28.1 | 0.74 (0.67, 0.81) |
| Family/friends | 64.9 | 1.92 (1.79, 2.05) |  | 26.6 | 1.72 (1.60, 1.85) |
| Support group | 66.6 | 1.45 (1.32, 1.58) |  | 25.9 | 1.04 (0.94, 1.15) |
| Hotline | 61.1 | 0.70 (0.62, 0.78) |  | 29.3 | 0.79 (0.70, 0.90) |
| Nurse/midwife/doula | 63.4 | 1.34 (1.24, 1.44) |  | 26.3 | 1.13 (1.05, 1.23) |
| Lactation specialist | 65.9 | 5.18 (4.83, 5.56) |  | 27.8 | 5.25 (4.86, 5.67) |
| Abbreviations: OR, odds ratio; CI, confidence interval  ^a^ Adjusted for age at birth, education, Kotelchuck index (prenatal care), race/ethnicity, and all other sources of breastfeeding information; reference group=did not breastfeed. | | | | | |

Supplemental Table 5. Effect of receiving breastfeeding information from a lactation specialist on breastfeeding <10 weeks and ≥10 weeks (compared to not breastfeeding), stratified by race/ethnicity, weighted to be site representative, Pregnancy Risk Assessment Monitoring System Phase 8, 2016-2019.

|  | **Breastfed**  **≥10 weeks** | **Breastfed**  **<10 weeks** |
| --- | --- | --- |
|  | **aOR (95% CI)^a^** | **aOR (95% CI)^a^** |
| **Race/ethnicity** |  |  |
| American Indian | 2.85 (2.13, 3.79) | 2.88 (2.15, 3.86) |
| Alaska Native | 2.44 (1.41, 4.24) | 2.79 (1.50, 5.19) |
| Asian | 3.98 (2.90, 5.46) | 3.47 (2.44, 4.95) |
| Black | 4.59 (3.93, 5.36) | 3.97 (3.38, 4.66) |
| Hispanic | 3.36 (2.80, 4.03) | 3.24 (2.66, 3.94) |
| White | 7.17 (6.52, 7.87) | 7.39 (6.65, 8.22) |
| Mixed race | 4.83 (3.36, 6.95) | 4.30 (2.86, 6.47) |
| Abbreviations: OR, odds ratio; CI, confidence interval  ^a^ Adjusted for age at delivery, education, prenatal care (Kotelchuck index), and all other sources of information (baby’s doctor, personal doctor, family/friends, support group, hotline, nurse/midwife/doula); associations within each racial/ethnic group were modeled separately; reference group=did not breastfeed | | |
